# Preservation solutions to improve graft patency: *The devil is in the detail*

**DOI:** 10.1186/s13019-020-01267-z

**Published:** 2020-08-27

**Authors:** Etem Caliskan, Catherine J. Pachuk, Louis P. Perrault, Maximilian Y. Emmert

**Affiliations:** 1 Department of Cardiovascular Surgery, Charité-Universitätsmedizin Berlin, Berlin, Germany; 2 Department of Cardiothoracic and Vascular Surgery, German Heart Center Berlin, German Heart Institute Berlin, Augustenburger Platz 1, 13353 Berlin, Germany; 3 Somahlution Inc, Jupiter, USA; 4 Department of Cardiac Surgery, Montreal Heart Institute, Université de Montréal, Montreal, Canada

We read with great interest the article by Fourquet and colleagues exploring whether autologous heparinized blood (AHB), heparinized saline (HS) and GALA (considered as their reference solution) have a protective effect on vein grafts interposed in the arterial position at 6 weeks post grafting in a syngeneic rat model [1].

The authors report significant intimal hyperplasia irrespective of treatment and further describe an endothelial-remodeling layer associated with an increase in wall thickness in each group at 6 weeks of follow-up. Based on these findings, they conclude that the storage solutions used in their experimental model lead to graft injury and that their reference solution (GALA) did not reduce risk of intimal hyperplasia. The study addresses an important topic and the experimental set up is interesting. However, there are several findings and conclusions that should be taken with caution and need further clarification.

First, we would like to point out that the solution used in the present study and referred to as GALA neither has the same composition as the GALA solution which was developed and used at the Veterans Affairs laboratories in Boston [2] nor is it comparable to the currently commercially available DuraGraft [3, 4]. In fact, it is to be recognized that the solution used by Fourquet and colleagues has an 11.4 mM ascorbic acid concentration which is 23-fold higher than the 0.5 mM ascorbic acid concentration used in the GALA solution or DuraGraft. Second, based on the potassium hydrogenophosphate concentration and the lack of corresponding phosphate acid, there appears to be no buffering capacity for this solution. Given the high concentration of ascorbic acid, this solution will be highly acidic, even in the presence of sodium bicarbonate. Additionally, ascorbic acid used in the concentrations such as described by Fourquet was demonstrated to be highly cytotoxic [5].

Next, with the exception of glucose (which also had a different concentration from GALA and DuraGraft), it is impossible to calculate concentrations of the other components as these were all listed as milliliters added of solutions with no concentrations associated with them.

Importantly, given the increase in ascorbic acid and the significant decreased buffering capacity, it must be assumed that the solution used in the present study had a very low acidic pH (i.e. close to pH 3 or lower). Therefore, this solution is extremely toxic to the endothelium of the implanted vein grafts. The high relevance of pH and proper buffering (to elevate the standard pH of saline from 5.5 up to 7.4) has been extensively described in the literature [6, 7] and has been highlighted in a sub-study of the PREVENT IV trial [8]. In this subanalysis, Harskamp et al. clearly demonstrated that the use of buffered saline was associated with a significantly lower occurrence of vein graft failure (VGF) when compared with NS or AWB [8].

For this reason, the use of a very acidic solution as used, Fourquet’s study would be unlikely to achieve the ultimate goal of endothelial protection thereby preventing the development of vein graft disease (VGD). On the other hand, their results are indeed consistent with those expected when vein grafts are stored in such a low pH acidic solution as used in their study.

Taking these significant differences in composition into account, any reference to the GALA solution or DuraGraft is inappropriate, and in our view, the conclusions made by Fourquet et al. that the GALA solution does not protect the endothelial layer when the vein graft was arterialized in this rat model is misleading and should be revised.

In particular, this is also due to the fact, that their findings are highly contradictory to the preclinical and clinical data available for the hospital compounded GALA solution and the commercially available DuraGraft (which was formulated based on the GALA solution) [2, 4, 9, 10].

In a recent retrospective analysis, Haime et al. showed that treatment of saphenous vein grafts with (the hospital compounded) GALA solution in patients undergoing isolated coronary artery bypass grafting was associated with a 45% lower occurrence of non-fatal MI, a 35% lower risk for revascularization and 19% lower major adverse cardiac events respectively starting at 1000 days post CABG [10]. Moreover, in a randomized multicenter clinical trial using a within-patient randomization design and sequential multidetector computed tomography angiography at 1, 3, and 12 months after CABG, Perrault et al. and colleagues found favorable effects on wall thickness (as a surrogate of VGF/VGD) of DuraGraft treated SVGs at 12 months, particularly in the proximal segments [4]. The study also showed a good safety profile for DuraGraft with very low rates of graft occlusion and occurrence of MI, and no deaths [4]. Finally, although the authors provided some limitations of their study, several important issues remain unaddressed. While they report graft thrombosis rates of 22% in the AHB group, 62.5% in the HS group and 82.5% in the GALA group respectively, these observations were not discussed in the context of one third of animals (3/9) in the GALA group dying during FU, making any meaningful interpretation and statistical analysis impossible. Last but not least, important technical details (i.e. storage time, size and diameter of the venous grafts, etc.) are not provided, but would however be mandatory to know for proper interpretation of the outcomes.

Taken together, while the study of Fourquet and colleagues addresses the important topic of graft patency, the results and drawn conclusions by the authors have to be taken with caution.

## Data Availability

Not applicable.
